# Nonparametric intensity bounds for the delineation of spatial clusters

**DOI:** 10.1186/1476-072X-10-1

**Published:** 2011-01-07

**Authors:** Fernando LP Oliveira, Luiz H Duczmal, André LF Cançado, Ricardo Tavares

**Affiliations:** 1 Statistics Department, Universidade Federal de Minas Gerais, Belo Horizonte, Brazil; 2 Ibmec, Minas Gerais, Brazil; 3 Statistics Department, Universidade de Brasília, Brasília, Brazil; 4 Mathematics Department, Universidade Federal de Ouro Preto, Ouro Preto, Brazil

## Abstract

**Background:**

There is considerable uncertainty in the disease rate estimation for aggregated area maps, especially for small population areas. As a consequence the delineation of local clustering is subject to substantial variation. Consider the most likely disease cluster produced by any given method, like SaTScan, for the detection and inference of spatial clusters in a map divided into areas; if this cluster is found to be statistically significant, what could be said of the external areas adjacent to the cluster? Do we have enough information to exclude them from a health program of prevention? Do all the areas inside the cluster have the same importance from a practitioner perspective?

**Results:**

We propose a method to measure the plausibility of each area being part of a possible localized anomaly in the map. In this work we assess the problem of finding error bounds for the delineation of spatial clusters in maps of areas with known populations and observed number of cases. A given map with the vector of real data (the number of observed cases for each area) shall be considered as just one of the possible realizations of the random variable vector with an unknown expected number of cases. The method is tested in numerical simulations and applied for three different real data maps for sharply and diffusely delineated clusters. The intensity bounds found by the method reflect the degree of geographic focus of the detected clusters.

**Conclusions:**

Our technique is able to delineate irregularly shaped and multiple clusters, making use of simple tools like the circular scan. Intensity bounds for the delineation of spatial clusters are obtained and indicate the plausibility of each area belonging to the real cluster. This tool employs simple mathematical concepts and interpreting the intensity function is very intuitive in terms of the importance of each area in delineating the possible anomalies of the map of rates. The Monte Carlo simulation requires an effort similar to the circular scan algorithm, and therefore it is quite fast. We hope that this tool should be useful in public health decision making of which areas should be prioritized.

## Background

There are many methods for the detection and inference of geographic clusters [1-10]. A large number of methods rely on the Spatial Scan Statistic [11], a development of the Naus spatial scan statistic [12]. Based on this statistic, several extensions were proposed, modifying the shape of the circular window used in the circular scan statistic [13] to include irregular shapes [14-20], see [21] for a recent review. However, those methods generally do not discuss the possible uncertainty in the delineation of the most likely cluster found.

There exists nowadays a crescent demand of interactive software for the visualization of spatial clusters [22]. A technique was presented [23] to visualize relative risk and statistical significance simultaneously. Given a map of *k *areas, with their respective centroids, the procedure builds a grid of equidistant points between all combinations of two, three and four adjacent area centroids. For each grid point the distances to the areas centroids are computed and sorted. These distances are used to define almost circular groupings of areas, with their respective cumulative numbers of observed and expected cases. The relative risk and the likelihood ratio are then calculated for each circular grouping. The likelihood ratio values are compared to the results of a Monte Carlo simulation under the null hypothesis that there are no clusters and the cases are uniformly distributed in the population, such that the expected number of cases in each area is proportional to its population. Groupings with likelihood ratios values exceeding 95% of those obtained from the simulation are stored and stratified into ten levels of relative risk. Within each risk level, the grouping with largest likelihood ratio is then mapped. Circular groupings with lower likelihood ratio are also mapped if they did not overlap any grouping previously mapped. The final result is a ten color shaded map of areas with statistically significant relative risks, providing a very effective visualization tool to grasp these two concepts.

A visual tool was developed [24] to find circular clusters using SaTScan, repeating the search for a set of *S *different values for the maximum cluster size parameter. The reliability of an area *i *is defined as the number of times this area is part of a significant circular cluster found by SaTScan, divided by the number *S*. A typical value of *S *is 8, with maximum-sizes ranging from 5% to 49%, as given in the paper. This approach allows the interactive visual identification the so-called "core clusters", which are loosely defined as those clusters which appear more consistently through the S multiple runs varying the maximum-size parameters. This method reveals additional information about the cluster structure, although restricted to the circular shape delineation imposed by formalism of the circular scan.

The program SaTScan [17] detects a spatial cluster in aggregated-area maps and compute its significance based on Monte Carlo simulations. This approach allows the characterization of a potential map anomaly, dividing the map into two areas, the cluster and the area outside it. In this work we are interested in pursuing further questions regarding the properties of individual areas inside and outside the detected cluster. We would like to assess the relative importance of individual areas within the cluster. We would also like to verify if the areas outside the cluster and adjacent to it could be indeed excluded from the suspected anomaly region in the map. These questions are important from a public health practitioner perspective. How to access quantitatively the risk of those areas, given that the information we have (cases count) is also subject to variation in our statistical modeling? A few papers have tackled these questions recently. For example, [25] produces confidence intervals for the risk in every area, which are compared to the risks inside the most likely cluster.

Geographic variability studies of disease rates are essential tools in etiology [26]. Maximum Likelihood Estimate Bayesian methods have been proposed to obtain unbiased rates, especially for rare diseases occurring in small population areas [27], thus providing more precise results than the usual maximum likelihood estimators (see [28]). This approach includes information from adjacent areas to locally estimate the risk, consequently reducing the quadratic mean error of the estimated rates. [29-31] approaches adjust the test significance levels for geographic risk excess. [32] proposed an empirical Bayes method employing Poisson likelihood with gamma prior in disease mapping. They also presented a non-parametric estimation for the prior using a method which is based on a spatial autoregressive procedure to model the prior distribution parameter devised by [33].

In this paper, we propose a different approach to delineate the "intensity bounds" associated to the most likely cluster, by running Monte Carlo simulations. The number of cases for each area is now considered as a random variable with mean equal to the observed rate, or to some smoothing function which takes into account its first order neighborhood. We will introduce a novel approach to assess the relative importance of individual areas in the composition of the clustering structure.

In our methodology we perform *m *Monte Carlo replications: we consider that the simulated number of cases for each area is the realization of a random variable with average equal to the observed number of cases of the original map. Then the most likely cluster for each replicated map is detected and the corresponding *m *likelihood values obtained by means of the *m *replications are ranked. For each area, we determine the maximum likelihood value obtained among the most likely clusters containing that area. Thus, we construct the *intensity function *associated to each area's ranking of its respective likelihood value among the *m *obtained values.

The main purpose of our method is to find the error bounds for the delineation of spatial clusters in maps divided into areas, through the definition of a criterion to measure the plausibility of each area being part of the cluster. As a by-product, our method is capable of identifying irregularly shaped clusters and multiple local clustering. This method is computationally fast and relies on basic ideas about the intrinsic variation of the observed number of cases for each area. This procedure allows the quantification of the uncertainty in the delineation of spatial clusters in a very precise and intuitive way, through the definition of the intensity function.

## Methods

### The Spatial Scan Statistics

The spatial scan statistic [11] considers a study area map *A *divided into *K *areas, with total population *N *and *C *total cases. A zone is any collection of connected regions. The null hypothesis assumes that there are no clusters and the cases are uniformly distributed in the population, such that the expected number of cases in each area is proportional to its population. And the number of cases in each region is Poisson distributed proportionally to its population. The number of observed cases is *c_z _*and the population is *n_z _*in the zone *z*. The expected number of cases under null hypothesis is given by *μ_z _*= *C*(*n_z_*/*N*). The relative risk of *z *is *I*(*z*) = *c_z_*/*μ_z _*and the relative risk outside *z *is *O*(*z*) = (*C *- *c_z_*)/(C - *μ_z_*). If *L*(*z*) is the likelihood function under the alternative hypothesis and *L*_0 _is the likelihood function under the null hypothesis, the logarithm of the likelihood ratio for the Poisson model is given by:

$$LLR(z)=\log\left(\frac{L\left(z\right)}{L_{0}}\right)$$

(1) $$LLR\left(z\right)=\{\begin{array}{ll} c_{z}\log\left(I\left(z\right)\right)+\left(C-c_{z}\right)\log\left(O\left(z\right)\right) & \text{if}\;I\left(z\right)>1\\ 0 & \text{otherwise} \end{array}$$

See [11] for details. *LLR*(*z*) is maximized over the chosen set *Z *of potential zones *z*, identifying the zone that constitutes the *most likely cluster*. When the set *Z *contain the zones defined by circular windows of different radii and centers, max_*z*∈*Z *_*LLR*(*z*) is the circular scan statistic. Other possible choices for *Z *includes elliptic and irregularly shaped clusters. The statistical significance of the most likely cluster of observed cases is calculated employing Monte Carlo simulation [34]. Under null hypothesis, simulated cases are distributed over the study area and the scan statistic is computed for the most likely cluster. This procedure is repeated many times, and the distribution of the obtained values is compared with the *LLR *of the most likely cluster of observed cases, producing its p-value.

### The intensity function

In this section we define a criterion to measure the plausibility of each area being part of a possible localized anomaly in the map. Instead of finding the most likely cluster in the original map with the observed number of cases for each area, we consider maps where the number of cases are replications of a vector of random variables, whose averages are defined based on the observed number of cases of the original map. We formalize this procedure in the following.

The original map has *c_i _*observed cases in the area *a_i_*, *i *= 1, ..., *K*. Now we construct a Monte Carlo replication randomly distributing the $C=\sum_{i=1}^{K}c_{i}$ cases among the *K *areas *a*_1_, ..., *a_K _*according to a multinomial distribution where the probability associated to the area *a_i _*is *c_i_*/*C*. Let *V *= (*s*_1_, ..., *s_K _*) the realization of the multinomial random vector where *s_i _*is the number of simulated cases in the area *a_i_*, *i *= 1, ..., *K*, where $\sum_{i=1}^{K}s_{i}=C$. The cluster finder algorithm (in our setting we use the circular scan) now finds the most likely cluster *MLC_1 _*with likelihood ratio value *LLR_1_*. The Monte Carlo procedure above is repeated *m *times, generating a set of *m *likelihood ratio values {*LLR*_1_, ..., *LLR_m_*} corresponding to the most likely clusters {*MLC*_1_, ..., *MLC_m_*}. The likelihood ratio values are sorted in increasing order as {*LLR*_(1) _, ..., *LLR*_(*m*)_} for the corresponding most likely clusters found {*MLC*_(1) _, ..., *MLC*_(*m*)_}. We now define the *intensity function f *: {1, ..., *m*} → ℝ by *f *(*j*) = *LLR*_(*j*)_, *j *= 1, ..., *m*.

For each area *a_i_*, let:

$$q\left(a_{i}\right)=\frac{1}{m}\;\;\underset{1\leq j\leq m,a_{i}\in MLC_{\left(j\right)}}{\arg\;\max}\;\;\;\;f\left(j\right),\;\;\;\;\;\;\;\;i=1,...,K.$$

If the area *a_i _*does not belong to any of the sets *MLC*_(1) _, ..., *MLC*_(*m*) _then we set *q*(*a_i_*) = 0. The value *q*(*a_i_*) represents the quantile of the highest likelihood ratio among the ranked values of the likelihood ratios of the most likely clusters found in the *m *Monte Carlo replications, which take into account the variability of the number of cases in each area. In this sense, the value *q*(*a_i_*) may be interpreted as the relative importance of the area *a_i _*as part of the anomaly of the map, where the value *f(j) *represents the maximum likelihood ratio found for the most likely clusters which contain the area *a_i_*. This concept gives more information about the anomaly than the clear-cut division between cluster and non-cluster areas, as given by the usual process of finding the most likely cluster in the original map.

### Rate correction using empirical Bayesian estimator

We shall consider a variation of the procedure described in the previous section. Instead of using the observed number of cases, this variant uses Marshall's smoothed estimates of the number of cases based on the information of first order neighborhood of each area. We then compute the intensity function in those two situations, employing the raw number of cases and Marshall's estimates.

Empirical Bayes methods were employed by [28] and [35]. Studies involving disease rates to show the geographical variability are common in epidemiological approaches. For this kind of approach it is important to assess the problem of obtaining unbiased estimates. Some Bayesian methods have been proposed in the literature for estimation of risks in small areas. These methods are based on information from other areas that comprise the region of study. One consequence of using these methods is to decrease the total mean square error of the estimates [27]. That is, relative risks are estimated more accurately by Bayesian methods than by using maximum likelihood estimation. Authors like [28] and [35] address this issue.

Using Bayesian methods in the estimation of spatial phenomena have the extra advantage of allowing the incorporation of spatial similarities between adjacent areas in risk estimates. Adding this information to the estimation of risk can lead to maps with more stable estimates and more precise differentiation between what is a true high (or very low) risk and what is indeed a random fluctuation caused by small populations. Moreover, it is expected that the estimates reproduce the spatial pattern of the real risks.

In our work we use the estimation procedure proposed by Marshall [28] to obtain estimates of relative risks. We use local empirical Bayesian estimators, because it is often reasonable to consider adjacent areas whose rates are similar because they are likely to be similar in other aspects. We use the first order neighbors of the area for which we want to get the estimated rate. The methodology developed by Marshall proposed an empirical Bayesian estimator for the risk of rare diseases, where one can approximate the distribution of the number of cases by the Poisson distribution, with parameter estimated by the method of moments. Consider a map divided into *k *areas indexed by *i*, *i *= 1, 2, ..., *k*. Suppose that events are recorded for each area in a period of time. Let *θ_i _*be the event rate in the *i*-th area and assume that *y_i_*, the number of events accumulated in the *i*-th area during this period, is distributed as a Poisson random variable with mean *E*(*y_i_*|*θ_i_*) = *n_i_θ_i_*, where *n_i _*is the population at risk in the *i*-th area. The maximum likelihood estimator of *θ_i _*is *t_i _*= *y_i_/n_i_*. This estimator has mean and variance conditioned on *θ_i _*given by *E*(*t_i_*|*θ_i_*) = *θ*_*i *_and *V*(*t_i_*|*θ_i_*) = *θ_i_/n_i_*, respectively. In the Bayesian approach, *θ*_*i *_has a *prior *distribution with mean $m_{i}=E_{\theta_{i}}$ and variance $a_{i}=V_{\theta_{i}}$. Unconditionally, *t_i _*has mean $m_{i}=E_{t_{i}}$ and variance $V_{t_{i}}=a_{i}+\frac{m_{i}}{n_{i}}$. Efron and *n*_*i *_Morris [27] showed that, given *m_i _*and *a_i_*, the best linear Bayes estimator for *θ_i _*is expressed by

$$\hat{\theta_{i}}=w_{i}t_{i}+\left(1-w_{i}\right)m_{i}$$

where $w_{i}=\frac{A_{i}}{\left(A_{i}+m_{i}/n_{i}\right)}$ is the a ratio between the a prior variance of *θ_i _*and the unconditional variance of *t_i_*. The global empirical Bayesian estimator proposed by [28] assumes that the distribution of *θ_i _*is the same for all areas and then replaces *m_i _*and *A_i _*by *m *and *A*, respectively. Using the method of moments, Marshall showed that the estimates for *m *and *A *are given, respectively, by $\tilde{m}=\frac{\sum y_{i}}{\sum n_{i}}$ and $\tilde{A}=s^{2}-\frac{\tilde{m}}{\bar{n}}$, where $s^{2}=\frac{\sum n_{i}\left(t_{i}-\tilde{m}\right)}{\sum n_{i}},\;\;\bar{n}=\frac{\sum n_{i}}{N}$ and *k *is the number of areas of the map. As the overall proposal is spatially invariant, i.e., independent of the performed permutation, the estimates do not change. It is necessary to change the expression of *θ_i _*for the estimation of the a prior parameters set to be performed based on information from the neighboring areas of *i*. In this case, *w_i_*, *m*, *s^2^*and *n *are replaced by *W_i_*, *M_i_*, $s_{i}^{2}$ and *n_i_*, respectively, calculated only with data from the neighboring areas of *i*, and are defined as the local empirical Bayesian estimators.

Marshall's smoothing procedure is advantageous when the number of cases is very small. It will be used for the Chagas' disease map, which has a reduced number of cases, as we shall see in the Results section.

## Results and Discussion

Our methodology was testing in numerical simulations and was applied in three case studies.

### Numerical Simulations

Three different types of "true" artificial clusters will be tested: a single circular cluster (in two maps with different relative risks), a L-shaped irregular cluster, and a double circular cluster (also in two maps with different relative risks). In all situations, the map consists of a rectangular array of 203 hexagonal cells, each cell with population 1000. The centroids of the hexagonal cells are not placed in a perfectly regular array; we introduced a slight random displacement on both *x *and *y *axes, in order to avoid ties when measuring distances between any two centroids. Cases are randomly distributed such that the cells inside the true cluster have higher probability of receiving cases than the areas outside it; the resulting maps with the randomly distributed cases are also displayed. That means that we will find clusters in "noisy" maps, where the number of cases is not homogeneously distributed inside and outside the artificial clusters. The clusters found by the circular scan are also shown. Finally, we display the resulting maps built through the intensity function.

### Single Circular Cluster

**Figure 1:**
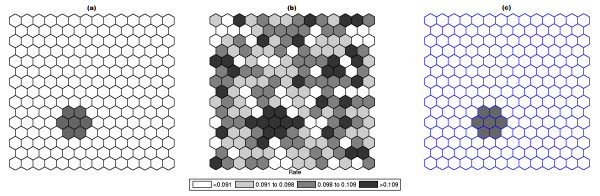
**A single circularly shaped true artificial cluster with very high relative risk (a), the random generated cases map of rates (b), and the cluster detected by the circular scan (c)**.

**Figure 2:**
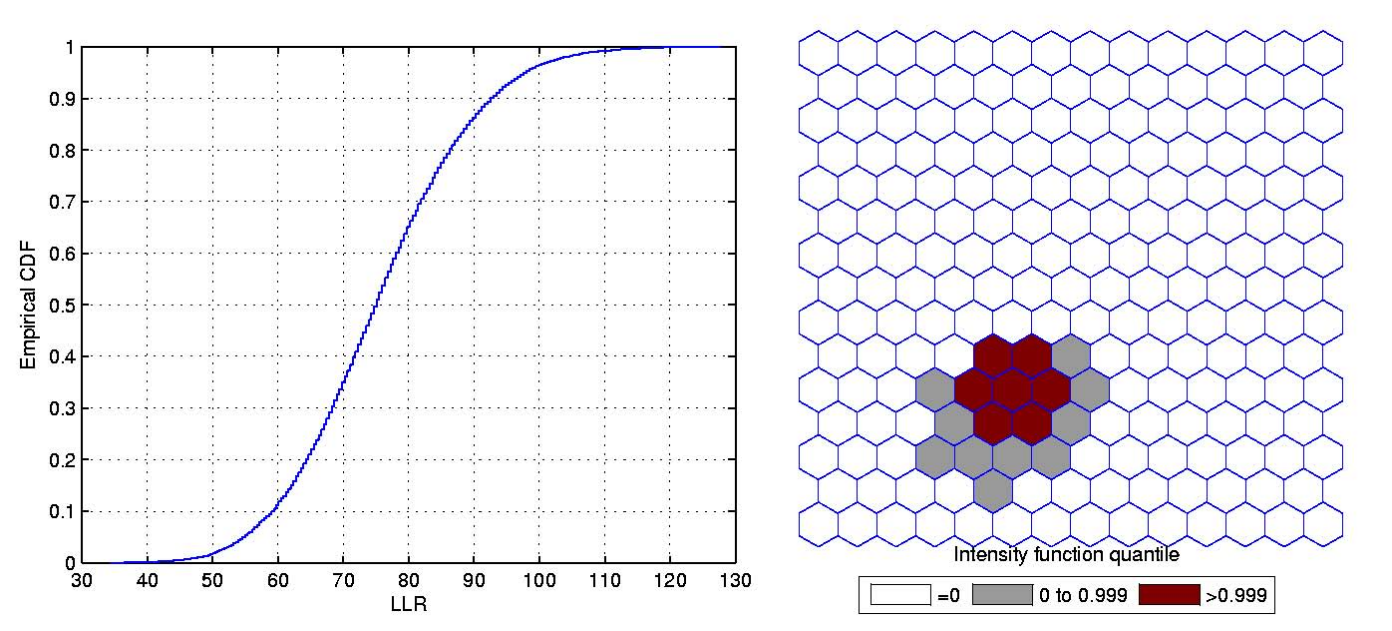
**The intensity function (a) and the intensity bounds map (b) for the very high relative risk single circular cluster**.

Figure 1 shows a circularly shaped true artificial cluster with very high relative risk (a), the random generated cases map of rates (b), and the cluster detected by the circular scan (c). The intensity function is displayed in Figure 2a. Finally, the intensity bounds map obtained by our method is shown in Figure 2b.

**Figure 3:**
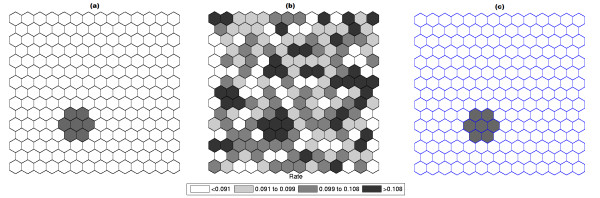
**A single circularly shaped true artificial cluster with moderately high relative risk (a), the random generated cases map of rates (b), and the cluster detected by the circular scan (c)**.

**Figure 4:**
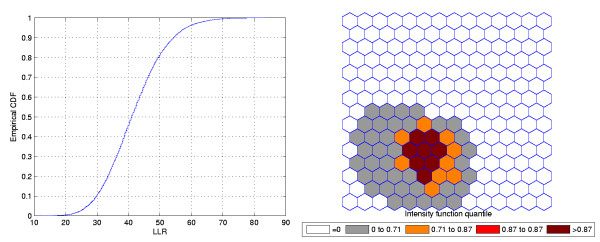
**The intensity function (a) and the intensity bounds map (b) for the moderately high relative risk single circular cluster**.

Figures 3 and 4 show the analogous results for another circularly shaped true cluster, with moderately high relative risk, for comparison.

The intensity bounds of the very high relative risk cluster are more sharply defined than those corresponding to the moderately high relative risk cluster, as expected. Observe that in both instances the true clusters were clearly detected, as represented by the darkest shade in Figures 2 and 4.

### Irregularly Shaped Cluster

**Figure 5:**
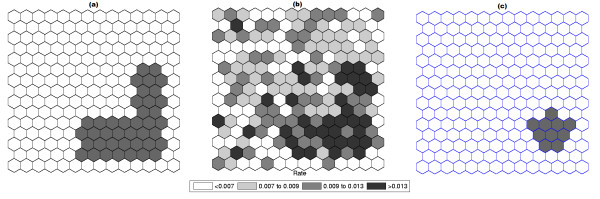
**The L-shaped true artificial cluster (a), the random generated cases map of rates (b), and the cluster detected by the circular scan (c)**.

**Figure 6:**
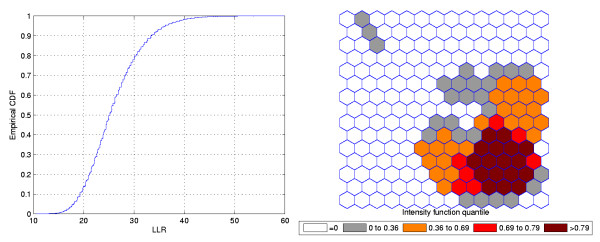
**The intensity function (a) and the intensity bounds map for the L-shaped artificial cluster**.

Figure 5 shows a L-shaped true artificial cluster (a), the random generated cases map of rates (b), and the cluster detected by the circular scan (c). The intensity function is displayed in Figure 6a. The intensity bounds map obtained by our method is shown in Figure 6b.

The circular scan detected a circular cluster centered in the angle formed by the two braces of the L-shaped cluster. However, the intensity bounds roughly delineated the L-shape, with a more intense region located around the angle of the L-shaped cluster. Sometimes the realizations of the random variable produced maps where circular clusters were found centered in the angle of the L-shaped cluster, but, very interestingly, also produced circular clusters centered along the braces of the L-shaped cluster. As a result, the overall intensity map of Figure 6 indicates the form of the L-shaped cluster.

### Double Circular Cluster

**Figure 7:**
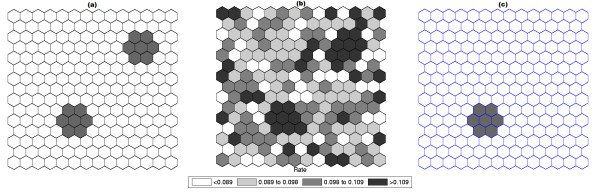
**A double circularly shaped true artificial cluster with very high relative risk (a), the random generated cases map of rates (b), and the cluster detected by the circular scan (c)**.

**Figure 8:**
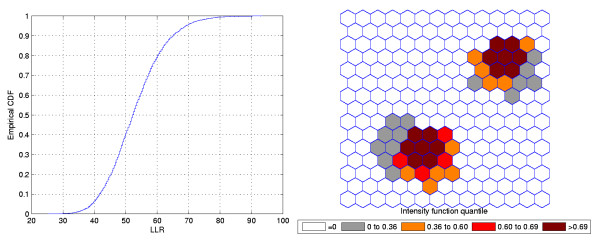
**The intensity function (a) and the intensity bounds map (b) for the double circularly shaped cluster with very high relative risk**.

Figure 7 shows a double circularly shaped true artificial cluster with very high relative risk (a), the random generated cases map of rates (b), and the cluster detected by the circular scan (c). The intensity function is displayed in Figure 8a. Finally, the intensity bounds map obtained by our method is shown in Figure 8b.

**Figure 9:**
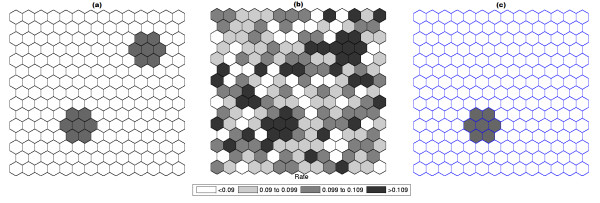
**A double circularly shaped true artificial cluster with moderately high relative risk (a), the random generated cases map of rates (b), and the cluster detected by the circular scan (c)**.

**Figure 10:**
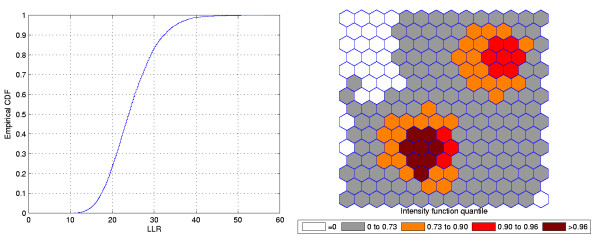
**The intensity function (a) and the intensity bounds map (b) for the moderately high relative risk double circular cluster**.

Figures 9 and 10 show the analogous results for another double circular true cluster, with moderately high relative risk, for comparison.

As displayed in Figure 7b and also in Figure 9b, the local rates of the two components of the double cluster are not equal, and the circular scan detected only the circular component cluster with the highest rate (Figures 7c and 9c). However, the intensity bounds delineated both circular clusters, with a more intense region located around the highest risk circular component (Figures 8b and 10b). Sometimes the realizations of the random variable produced maps where the highest risk circular component was found, but also produced circular clusters centered in the lower risk component. As a result, the overall intensity map indicates the two components, with different intensities.

### Real Data Case Studies

To illustrate our method, we present three real data case studies. In the first study, with homicide cases in Minas Gerais state, Brazil, the most likely cluster is compact and very sharply delineated, being highly geographically focused. The second study is a well-known benchmark of female breast cancer in the Northeast U.S. [11], and the third case study displays Chagas' disease cases in puerperal women, also in Minas Gerais state, Brazil. In those two last studies, the most likely clusters are not sharply delineated, being moderately geographically focused. The breast cancer study has many cases, compared to the reduced number of cases of the Chagas' disease study, allowing us to compare the performance of the map in two very different situations.

In the Chagas' disease study we used both the raw and Marshall's smoothed rates, due to the small number of cases. On the other hand, for the the other two studies we have only presented raw rates results, because there are no advantages in employing smoothed rates when the raw rates are based in a large number of cases. For all maps, each area *a_i _*will be colored according to the quantile given by the function value *q*(*a_i_*), as explained in the previous section. The choice of the quantile level representation by distinct shades of color varies in each map. We have chosen quantile levels in order to improve the visualization of the intensity function in the maps. All blank areas were never part of any cluster in the Monte Carlo simulations, corresponding to those areas *a_i _*for which *q*(*a_i_*) = 0. In the software, the user may choose arbitrary quantiles to represent the data. All the programming was made using Matlab 7.10 and the code is available from the authors.

#### Homicide Clusters

Minas Gerais state is located in Brazil's Southwest and consists of 853 municipalities, with 20,912 registered homicides from 2003 to 2007, and an estimated population of 19,150,344 in 2005. Data are available from the Brazilian Ministry of Health http://www.datasus.gov.br and the Brazilian Institute of Geography and Statistics http://www.ibge.gov.br.

**Figure 11:**
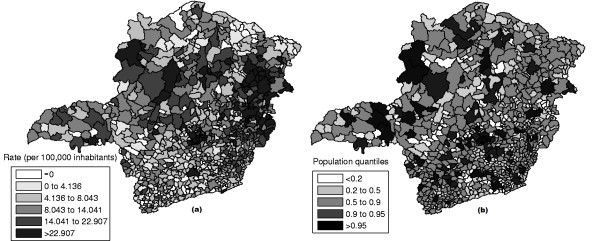
**Homicide rates map (a) and population at risk map (b) in Minas Gerais State, Brazil**.

**Figure 12:**
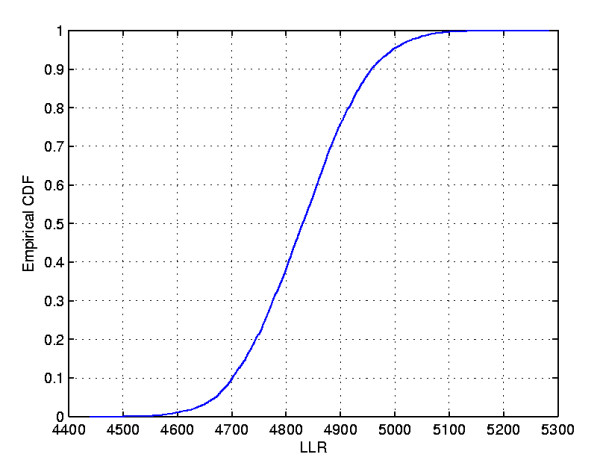
**The intensity function for the homicides map**.

**Figure 13:**
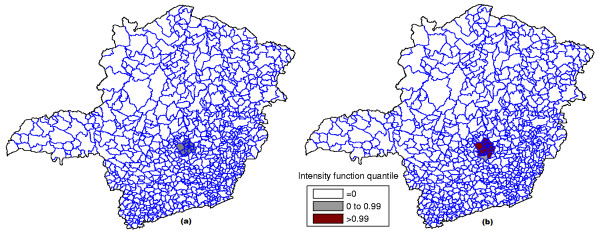
**The most likely cluster found by the circular scan (a) and intensity function map (b) for the homicides map**.

The raw rates map is presented in Figure 11a and the population at risk map in Figure 11b. The Monte Carlo procedure described in the Methodology section is performed for the raw rates, producing their respective intensity function. The intensity function for the raw rates map is displayed in Figure 12. Figure 13a shows the most likely cluster found by circular scan. Figure 13b show the map corresponding to the intensity function derived from the raw rates map.

In the intensity function map, the non-blank areas attain almost the same level, meaning that the anomaly is very conspicuous. On the other hand, this anomaly is compact and coincides with the most likely cluster found by the circular scan. Although there are other places in the map where the rates are elevated, the values of the intensity function are not elevated enough to produce non-blank areas outside the anomaly in the center of the map.

#### The Breast Cancer Clusters in Northeastern United States

The data set of mortality from breast cancer in the Northeastern U.S. consists of age-adjusted 58,943 deaths for the period from 1988 to 1992, with the female population at risk of 29,535,210 in 1990. This map consists of 245 counties in 10 states and the District of Columbia. This dataset has been studied in detail using the circular spatial scan statistic [36] and the elliptic spatial scan statistic [37].

**Figure 14:**
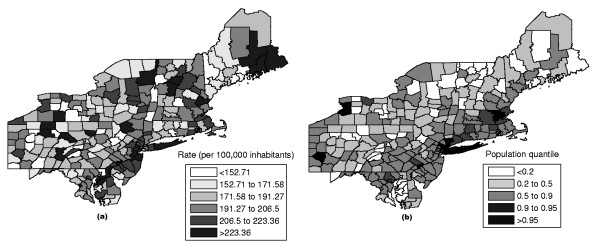
**The rates map (a) and population at risk map (b) for the Northeast U.S. breast cancer data**.

**Figure 15:**
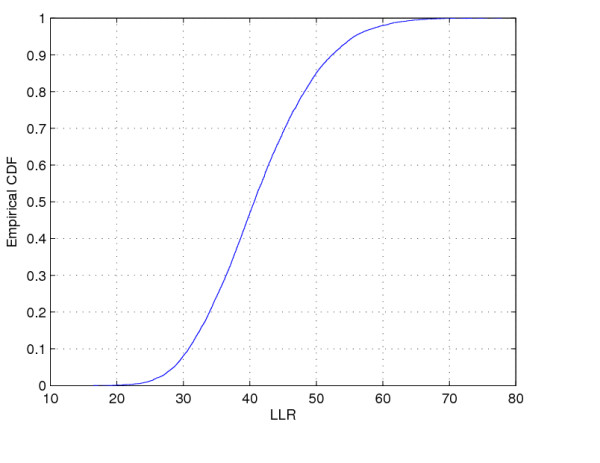
**The intensity function for the Northeast U.S. breast cancer data**.

The raw rates map is presented in Figure 14a and the population at risk map in Figure 14b. The Monte Carlo procedure is performed producing its respective intensity function, displayed in Figure 15.

**Figure 16:**
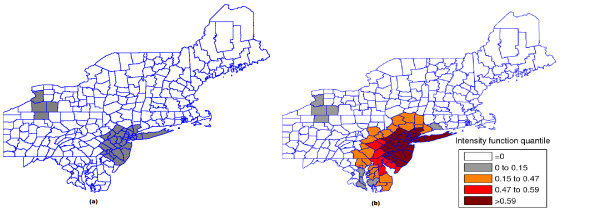
**The three strongest clusters found by SaTScan **[36]** (a) and intensity function map (b) for the Northeast U.S. breast cancer data**.

This case study presents a very different situation from the first example. The map derived from intensity function in Figure 16b shows the presence of various anomalies placed at different parts of the study area, indicating their geographic focus. We clearly observe three distinct groups of shaded areas in Figure 16b, consistently matching with the three strongest clusters found by SaTScan [36], shown in Figure 16a. The darkest shaded group is associated to the New York, NY-Philadelphia, PA primary cluster, with p-value 0.0001. The upper left group of four gray areas coincides exactly with the Buffalo, NY secondary cluster, with p-value 0.122. Finally the gray area at the lower center of the map corresponds to the Washington, DC secondary cluster, with p-value 0.147.

This example shows that the intensity function has the ability to delineate even the multiple and irregularly shaped potential clusters. We stress the fact that, for each Monte Carlo replication, only the primary most likely cluster was used to build the map derived from the intensity function of Figure 16b.

#### Chagas' Disease Clusters

This subsection presents the data set of Chagas' disease cases in puerperal women in Minas Gerais state, Brazil. The population at risk consists of women that gave birth to babies in the period of July to September, 2006. The new-born babies were blood tested to detect the presence of the Chagas disease antigen, with coverage above 96%. A positive test means that the mother is infected. These tests were conducted through the project PETN-MG (Minas Gerais State Program of New-Born Screening) coordinated by the research group NUPAD-MEDICINA/UFMG from Federal University of Minas Gerais Medical School http://www.nupad.medicina.ufmg.br in collaboration with Minas Gerais State Health Secretary. The state is divided into 853 municipalities with a total population at risk of 24,969 women. After a comprehensive screening to eliminate false positives a total number of 113 cases were obtained.

**Figure 17:**
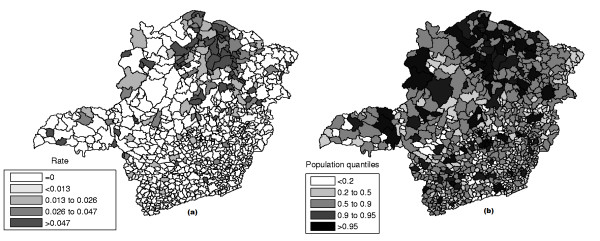
**Chagas' disease rates map (a) and population at risk map (b) in Minas Gerais State, Brazil**.

**Figure 18:**
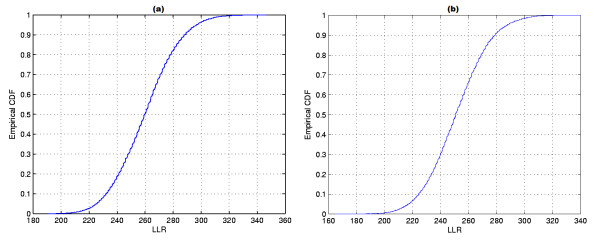
**The intensity functions of the raw rates (a) and smoothed rates (b) for the Chagas' disease map**.

**Figure 19:**
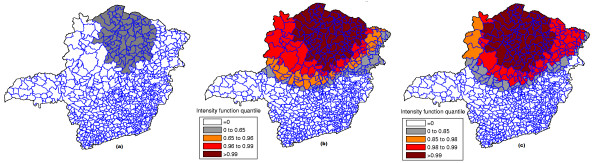
**The most likely cluster found by the circular scan for the raw rates map (a), the raw rates intensity function map (b) and Marshall's smoothed rates intensity function map (c) for the Chagas' disease map**.

The raw rates map is presented in Figure 17a and the population at risk map in Figure 17b. The Monte Carlo procedure is performed for both the raw rates and Marshall's smoothed rates maps, producing their respective intensity functions. The intensity function for the raw rates map is displayed in Figure 18a. The intensity function for Marshall's smoothed rates is displayed in Figure 18b. Figure 19a shows the most likely cluster found by circular scan. Figures 19b and 19c show the maps corresponding to the intensity function derived from the raw rates map and the smoothed rates map, respectively.

The maps derived from the raw (Figure 19b) and smoothed (Figure 19c) intensity functions show the presence of a strong anomaly. For the map of Figure 19b, the area formed by the highest intensity areas (dark colored) coincides almost perfectly with the primary cluster found by the circular scan. However, the corresponding area of Figure 19c does not match so well the primary cluster, due to the overdispersion created by Marshall's smoothing procedure. In both maps, we observe that the anomaly is less geographically focused, spreading over the northern part of the state. This example shows that the error bounds of the existing cluster were easily visualized by means of the intensity function. The application of Marshall's smoothing procedure does not contribute to improve the delineation of the anomaly, even considering that there are few cases in the study area.

## Conclusions

Our methodology takes into account the variability in the observed number of disease cases on area-aggregated maps to nonparametrically infer the uncertainty in the delineation of spatial clusters. A given real data map is regarded as just one possible realization of an unknown random variable vector with expected number of cases. The real data vector of the number of observed cases in each area is used to construct a new vector of expected values of random variables, either as a composition of neighboring areas in the map, employing Marshall's smoothing, or either considering the raw count of cases as the average of the random variables. This vector is now an estimate of the unknown random variable vector with expected number of cases. Our methodology performs *m *Monte Carlo replications based on this estimated vector of averages. The most likely cluster of each replicated map is detected and the *m *corresponding likelihood values obtained in the replications are ranked. For each area we determine the maximum likelihood value among the most likely clusters containing that area. Thus, we obtain the intensity function associated to each area's ranking of their respective likelihood value among the *m *values. The intensity of each area can be interpreted as the importance of that area in the delineation of the possibly existing anomaly on the map, considering only the initially given information of the observed number of cases. This procedure, based on empirical distribution, takes into account the intrinsic variability of the observed number of cases, which generally is not considered directly in the existing algorithms used to detect spatial clusters.

In our case studies we could see different situations with respect to the intrinsic variability of the existing spatial anomaly. When the most likely cluster is quite prominent, as seen in the homicides map example, the intensity function is such that almost all areas associated with the most likely clusters found in the *m *replications coincides with those areas composing the most likely cluster detected for the original observed cases. In this example the geographic anomaly is highly focused. However, in the other two case studies, the opposite happens. The Chagas' disease map presents an intrinsically wide variability of data. Many areas near or adjacent to the most likely cluster have values of the intensity function close to the values corresponding to areas of the most likely cluster. In the case study of breast cancer, this intrinsic variability produces a map with clearly unrelated areas, but with rather close probability ranking, indicating a situation of multiplicity of clusters, i e., the most likely cluster is clearly poorly delineated. It is noteworthy that the entire procedure was performed using the circular scan, and even then it identifies irregular and multiple clusters.

An analogy with our proposed method can be found in image analysis: suppose we take several short digital exposures of a very low light level scene, e.g. some deep-sky field of galaxies. Each exposure generates an image consisting of a rectangular matrix of pixels, each pixel receiving a small number of photons corresponding to the illumination of its small associated portion of the image. The expected rate of photons is constant during all the exposures, but the number of photons received by the same pixel varies from one exposure to the other due to the stochastic nature of the process. Usually, one simply adds the values for the same pixel through all the exposures, to compose a single final image with higher sharpness (signal-to-noise). Instead, we first submit each exposure image through a filter, which in our case is the algorithm to detect the most likely cluster, and then compose all the corresponding clusters into a single "cluster image" by means of the intensity function. If the "real" cluster is very contrasting with the background noise, all exposures will produce very similar clusters, thus producing a sharply defined final cluster image. Otherwise, when the real cluster is not very conspicuous, we should observe a large variation in individual clusters, producing a poorly delineated cluster in the final image.

We presented two variants of the computation of the intensity function. The first employed the raw number of cases, and the second used Marshall's smoothed estimates of the number of cases based on the information of the first order neighborhood of each area. This was done because we were especially concerned with areas containing zero cases, which could generate biased Monte Carlo distributions of cases over the map. Marshall's smoothed estimates of cases could potentially alleviate this problem providing non-zero averages employed in the multinomial random vector. However, we have noted in all our examples that the application of Marshall's smoothed estimates produces less sharply defined intensity function maps, compared to those obtained by the use of the raw cases data. On the other hand, we could not observe any artifacts due to the use of non-smoothed raw cases data in the delineation of the anomaly. This may be explained by the simple fact that the circular spatial scan works itself as a "filter", when it joins several areas within the circular window, thus naturally diminishing the effect of the zero cases areas in the composition of the cluster candidates. This suggests that the utilization of raw cases data does not seem to interfere with the visualization of the intensity bounds.

This tool uses simple mathematical concepts and the interpretation of the intensity function *f *is very intuitive in terms of the importance of each area in delineating the possible anomalies of the map of rates.

The Monte Carlo simulation requires an effort similar to the circular scan algorithm, and therefore it is quite fast. Furthermore, the accuracy of the interactive construction of the map from the intensity function *f *increases gradually with execution time. Thus the user could stop the simulation process at any time when it is realized that the delineation of potential anomalies will converge. We therefore hope that this tool may assist in the decision process of prioritizing the areas of a map associated with potential spatial anomaly.

## Competing interests

The authors declare that they have no competing interests.

## Authors' contributions

FLPO, LHD and ALFC proposed the methodology, developed the programs, conducted the simulations, analyzed the case studies and drafted the manuscript. RT contributed with the implementation of Marshall's smoothing procedure. All authors read and approved the final manuscript.
